# Shiga toxin-producing *Escherichia coli* in beef retail markets from Argentina

**DOI:** 10.3389/fcimb.2012.00171

**Published:** 2013-01-18

**Authors:** Victoria Brusa, Virginia Aliverti, Florencia Aliverti, Emanuel E. Ortega, Julian H. de la Torre, Luciano H. Linares, Marcelo E. Sanz, Analía I. Etcheverría, Nora L. Padola, Lucía Galli, Pilar Peral García, Julio Copes, Gerardo A. Leotta

**Affiliations:** ^1^Laboratorio de Microbiología de Alimentos, Instituto de Genética Veterinaria “Ing. Fernando N. Dulout”, Facultad de Ciencias Veterinarias, Universidad Nacional de La Plata, CCT-La Plata, CONICETBuenos Aires, Argentina; ^2^Inmunoquímica y Biotecnología, CIVETAN, CONICET-CICPBA, Facultad de Ciencias Veterinarias, Universidad Nacional del Centro de la Provincia de Buenos AiresBuenos Aires, Argentina

**Keywords:** STEC, raw ground beef, retail markets, environmental samples, Argentina

## Abstract

Shiga toxin-producing *Escherichia coli* (STEC) are foodborne pathogens that cause mild or serious diseases and can lead to people death. This study reports the prevalence and characteristics of STEC O157 and non-O157 in commercial ground beef and environmental samples, including meat table, knife, meat mincing machine, and manipulator hands (*n* = 450) obtained from 90 retail markets over a nine-month period. The STEC isolates were serotyped and virulence genes as *stx* (Shiga toxin), *rfb*_O157_] (O157 lipopolysaccharide), *fli*C_H7_ (H7 flagellin), *eae* (intimin), *ehx*A (enterohemolysin) and *saa* (STEC autoagglutinating adhesin), were determined. STEC O157 were identified in 23 (25.5%) beef samples and 16 (4.4%) environmental samples, while STEC non-O157 were present in 47 (52.2%) and 182 (50.5%), respectively. Among 54 strains isolated, 17 were STEC O157:H7 and 37 were STEC non-O157. The prevalent genotype for O157 was *stx*_2_/*eae*/*ehx*A/*fli*C_H7_ (83.4%), and for STEC non-O157 the most frequent ones were *stx*_1_/*stx*_2_/*saa*/*ehx*A (29.7%); *stx*_2_ (29.7%); and *stx*_2_/*saa*/*ehx*A (27%). None of the STEC non-O157 strains were *eae*-positive. Besides O157:H7, other 20 different serotypes were identified, being O8:H19, O178:H19, and O174:H28 the prevalent. Strains belonging to the same serotype could be isolated from different sources of the same retail market. Also, the same serotype could be detected in different stores. In conclusion, screening techniques are increasingly sensitive, but the isolation of STEC non-O157 is still a challenge. Moreover, with the results obtained from the present work, although more studies are needed, cross-contamination between meat and the environment could be suspected.

## Introduction

Foodborne illnesses are defined as diseases, usually either infectious or toxic in nature, caused by agents that enter the body through the ingestion of food. Shiga toxin-producing *Escherichia coli* (STEC) are a group of food and water-borne pathogens associated with a wide spectrum of human diseases, ranging from mild diarrhea to hemorrhagic colitis (HC), thrombocytopenia, hemolytic uremic syndrome (HUS), and can also lead to people death (Karmali et al., 2010). Argentina has a high incidence of HUS: 17 cases per 100,000 children younger than 5 years, one of the highest in the world (Rivas et al., 2010). This rate is 10-fold higher than in other industrialized countries (Mead and Griffin, 1998). In Argentina, STEC is the primary etiological agent of HUS (Leotta et al., 2008), and *E. coli* O157:H7 is the predominant serotype isolated. However, other serotypes were associated with HUS (Guth et al., 2011).

Ruminants have been identified as the major reservoir of STEC (Karmali et al., 2010) and a variety of foods have been identified as vehicles of illnesses. However, approximately 52% of outbreaks have been associated with bovine products. Contamination of carcasses with STEC can occur when gut contents or fecal matter contact the meat surfaces, also cross-contamination between carcasses may occur during processing (Edwards and Fung, 2006). Studies of STEC prevalence in feces and carcasses of bovines from selected beef exporting abattoirs of Argentina showed that STEC non-O157 was present in 22.3% and in 9.0% of the feces and bovine carcasses (Masana et al., 2011), and the prevalence of STEC O157 was 4.1% and 2.6%, respectively (Masana et al., 2010). In other study 12.34% and 18.64% of STEC in carcasses, were detected at the slaughter and sanitary control cabin, respectively. These percentages increased at butcheries (24.52%) from the same city. The 25% of retail beef cuts were STEC-positive with significant differences among the different cuts of meat (chuck: 12.12%, rump roast: 12.12% and minced beef: 40.74%) (Etcheverría et al., 2010). Comparatively, the prevalence of STEC non-O157 in the USA beef cattle feces has been shown to range from 19 to 30% (Barkocy-Gallagher et al., 2003; Renter et al., 2005) and the prevalence in hides was 56.3% (Barkocy-Gallagher et al., 2003).

Some studies of STEC prevalence in minced beef samples were made in Argentina, in one of them, 3.8% of O157:H7 was detected (Chinen et al., 2001) while Parma et al. (2000) found serotypes mainly non-O157. Studies in France, Australia, and the USA determined a STEC prevalence of approximately 16% in minced beef samples (Bohaychuk et al., 2006), while in Spain (Mora et al., 2007) the prevalence was 12%.

Until present, there have not been systematic studies on local ground beef retail markets to assess the microbiological quality of meat, but also including verification of good hygiene practices, handlers' habits and traceability of the raw material. In Argentina, the microbiological quality of the meat sold at retail markets is based on the parameters specified at the Argentine Food Code. However, for STEC strains only O157:H7 serotype is mandatory, and the searching for potentially pathogenic bacteria for consumers on surfaces that contact with the meat is not established.

The aims of this study were the detection, isolation, and characterization of STEC strains in ground beef and environmental sponge samples, including meat table, knife, meat mincing machine, and manipulator hands, in ground beef retail markets, and to determine the pathogenic potential of the circulating strains. It also attempts to establish potential cross-contamination throw surfaces.

## Materials and methods

### Sample collection

From October 2010 to July 2011, 450 samples (90 raw ground beef and 360 environmental samples) were weekly collected from 90 retail stores in Berisso city, Buenos Aires province, Argentina. Sampling collection was randomly performed and covered all the geographic areas of the city. Environmental samples were obtained from meat contact surfaces such as meat tables, knives, meat mincing machines, and manipulator hands, following the protocol described below. From meat tables, three 400 cm^2^ areas were sampled with a sterile sponge soaked in buffered peptone water (Biokar, Zac de Ther, France). The entire surface of the knife blade and the intersection between the blade and the blade handle were sponged. The meat mincing machine was disassembled and the sample was taken from the meat container, the worm meat grinder, and the screw ring. Both hands (front, back, and nails) of the manipulator were sampled. All samples were ice refrigerated and sent to the laboratory to be analyzed immediately.

### Culture enrichment, PCR assay, and isolation

The samples were analyzed by duplicate. One replicate was processed according to US Department of Agriculture, Food Safety and Inspection Service (FSIS) methodology MLG 5.05 for *E. coli* O157:H7 (USDA-FSIS, 2010), and the other for STEC screening. Briefly, a 65 g portion of raw ground beef was placed aseptically in a plastic bag with 585 mL of modified tryptic soy broth (mTSB) with 20 mg/L of novobiocin and casaminoacids (Acumedia Manufacturers, USA). To the sponges 100 ml of mTSB with 20 mg/L novobiocin plus casaminoacids were added. After homogenizing in a stomacher (Interscience, Saint Nom, France), each sample was incubated for 15–22 h at 42°C. All the samples were processed by immunomagnetic separation (IMS) (Dynal Biotech, Oslo, Norway) according to the manufacturer's instructions, and plated onto CT-SMAC (Oxoid, Basingstoke, Inglaterra) and SD39 (Acumedia). Suspect colonies were screened for *stx*_1_, *stx*_2_, and *rfb*_O157_ genes by multiplex PCR (Leotta et al., 2005). For STEC screening 25 g of raw ground beef and environmental samples were incubated at 41.5°C for 15–22 h in 225 mL modified *E. coli* broth (mEC) (Acumedia) and 90 mL of mEC (Acumedia), respectively. One milliliter of the broth culture was taken for DNA extraction, using triton 1% in TE 1×, to be analyzed by an intralaboratory validated SYBR-Green real time PCR analysis (Brusa et al., 2011). The PCR was performed in a 20 μl reaction mixture containing 4 μl of DNA template, 10 μl of PerfeCTa SYBR Green SuperMix, low ROX (Quanta, Biosciences), and 0.2 μl of 100 μM of each primer. Primer sequences were: stx1-F GCAGATAAATCGCCATTCG, stx1-R TGTTGTACGAAATCCCCTCTG, stx2-F CATGACAACGGACAGCAGTTA, and stx2-R TGTGGATGCATCTCTGGTCA (Operon, Huntsville, AL, USA). Thermal cycle condition was as follows: 1 cycle at 95°C for 10 min, 40 cycles at 95°C for 10 s, and 56°C for 30 s, followed by a cycle at 95°C for 1 min, 55°C for 30 s, and 95°C for 30 s. *E. coli* O157:H7 EDL933 and *E. coli* K-12 were used as positive and negative controls, respectively. One milliliter of PCR-positive samples for *stx*_1_ and/or *stx*_2_ genes were spin down and the pellet was plated onto MacConkey agar (MAC) (Becton Dickinson Co., Sparks, MD, USA) and subsequently, in three consecutive eosin-methylene blue–Levine (Biokard, Zac de Ther, France) agar plates and incubated at 37°C for 18 h. The confluent growth zone onto MAC agar was screened for *stx*_1_ and *stx*_2_ genes by multiplex PCR (Leotta et al., 2005). At least 30 presumptive *E. coli* colonies were selected from any *stx*-positive plate for PCR confirmation. STEC strains were isolated in Trypticase soy agar (TSA) (BD Co.), confirmed by multiplex PCR (Leotta et al., 2005), and stored in Trypticase soy broth (TSB) with 30% and 40% glycerol at −20°C and −70°C, respectively, for further phenotypic and genotypic characterization.

### Phenotypic and genotypic characterization of isolates

Confirmation of isolates as *E. coli* was performed through biochemical tests according to Ewing and Edwards (1986). In all STEC O157 isolates, *stx*_1_, *stx*_2_, and *rfb*_O157_ genes were detected by multiplex PCR as described above, while the *eae* (intimin), *ehx*A (enterohemolysin), and *fli*C_H7_ (H7 flagellin) genes were investigated as described by Karch et al. (1993), Schmidt et al. (1995), and Gannon et al. (1997), respectively.

Virulence factors for STEC non-O157 isolates were detected as previously described for O157, and also *saa* (autoagglutinating adhesin) gene was investigated in intimin-negative strains (Toma et al., 2004). The O and H antigens were determined by a microagglutination technique in plates and by tube agglutination technique, respectively, with an antisera kit (O1-O186) and 56 H antisera produced by Laboratorio de Referencia de *E. coli* (LREC) (Lugo, Spain), as described by Blanco et al. (1997).

## Results

### Prevalence of O157:H7 and non-O157 STEC strains

Among the 90 raw ground beef samples and 360 environmental samples, 25.5% beef and 4.4% environmental samples, were positive for STEC O157:H7 while 52.2% beef and 50.5% environmental samples were positive for non-O157 STEC strains, by the PCR screening (Table 1). From the 90 meat tables, 90 knives, 90 mincing machines, and 90 manipulator hands analyzed, 2.2, 3.3, 6.6, and 5.5%, respectively, were positive for STEC O157:H7; while, 55.5, 46.6, 61.1, and 38.8%, respectively, were positive for STEC non-O157 (Table 1). Overall, STEC O157:H7 were isolated from 11/23 (47.8%) raw ground beef samples and 6/16 (37.5%) environmental samples (*n* = 2 meat table, *n* = 1 knife, *n* = 1 mincing machine, *n* = 2 manipulator hands) (Table 1). STEC non-O157 were isolated from 13/47 (27.6%) raw ground beef samples and 24/182 (13.2%) environmental samples (*n* = 9 meat table, *n* = 7 knife, *n* = 6 mincing machine, *n* = 2 manipulator hands) (Table 1).

**Table 1:** **Number of STEC PCR-positives samples and strains isolated from different sources**.

**Sample category**	**No. of samples analyzed**	**No. (%) of STEC O157 PCR positive samples**	**No. (%) of STEC O157 strains isolated**	**No. (%) of STEC non-O157 PCR positive samples**	**No. of STEC non-O157 strains isolated**
Raw ground beef	90	23 (25.5)	11 (47.8)	47 (52.2)	13 (27.6)
Meat tables	90	2 (2.2)	2 (100)	50 (55.5)	9 (18)
Knives	90	3 (3.3)	1 (33.3)	42 (46.6)	7 (16.6)
Mincing machine	90	6 (6.6)	1 (16.6)	55 (61.1)	6 (10.9)
Manipulator hands	90	5 (5.5)	2 (40)	35 (38.8)	2 (5.7)

### Characterization of O157 and non-O157 STEC isolates

Fifty-four STEC isolates (*n* = 17 O157, *n* = 37 non-O157) were characterized by phenotypic and genotypic techniques, and then serotyped. STEC O157 characterization proved that all isolates were sorbitol and cellobiose negatives. β-glucuronidase negative and Biotype C (rhamnose + / rafinose + / dulcitol +) was predominant, with only two strains belonging to biotype D (rhamnose + / rafinose + / dulcitol −). All STEC O157 isolates harbored *eae*, *ehx*A, and *fli*C_H7_ genes, while 83.4% were *stx*_2_, and 16.6% were *stx*_1_/*stx*_2_-positive. Although all O157 were *fli*C_H7_-positive, 6% were non-motile.

All STEC non-O157 strains were sorbitol and ortho-Nitrophenyl-β-galactoside (ONPG) positives. Most of the non-O157 strains were β-glucuronidase positive (73%), and motile (97.3%). Among 37 STEC non-O157 isolates, 20 different serotypes were identified, comprising 12 O serogroups (O8, O41, O44, O49, O79, O113, O116, O130, O171, O174, O178, and O181) and 8 H antigens, being H19 (*n* = 16) and H21 (*n* = 6) the prevalent ones (Table 2). Five strains were O-non-typeable and two strains H-non-typeable. The most prevalent serotypes were O8:H19 and O178:H19 (13.5%); O174:H28 (10.8%); O41:H14, O79:H19, O113:H21, O174:H21, and O181:H49 (5.4%). Other serotypes were isolated just one time (Table 2).

**Table 2:** **Serotypes, source, Shiga toxin genotypes and other virulence markers of the strains studied**.

**Serotype**	**No. of strains**	**Sources**					**Virulence markers**				
		**GB**	**MT**	**K**	**MM**	**MH**	***stx*_1_**	***stx*_2_**	***ehx*A**	***saa***	***eae***
O157:H7	17	1	1		1		+	+	+	−	+
		10	1	1		2	−	+	+	−	+
O8:H19	5	1	2	1			+	+	+	+	−
		1					−	+	+	−	−
					1		−	+	+	+	−
O41:H14	2	2					−	+	−	−	−
O44:NT HNM	1		1				−	+	−	−	−
O49:H49	1		1				−	+	+	+	−
O79:H19	2	1	1				−	+	+	+	−
O113:H21	2	1			1		+	+	+	+	−
O116:H21	1			1			−	+	+	+	−
O130:H21	1			1			+	+	+	+	−
O171:H2	1	1					+	+	−	−	−
O174:H–	1					1	−	+	−	−	−
O174:H8	1	1					+	−	−	−	−
O174:H21	2	1	1				−	+	−	−	−
O174:H28	4		2	1	1		−	+	+	+	−
O178:H19	6	1	1	1	2		−	+	−	−	−
O181:H49	2	1	1				+	+	+	+	−
ONT:H18	1				1		+	+	+	+	−
ONT:H19	3	1					+	+	+	+	−
		1					+	+	+	−	−
		1					−	+	+	−	−
ONT:HNM	1			1			−	+	+	+	−

GB, ground beef; MT, meat table; K, knife; MM, mincing machine; MH, manipulator hands; ONT, O-untypeable; HNM, non-motile strains; NT, H-untypeable.

Different *stx* genotypes occurred among non-O157 strains (Table 2). The frequency of *stx* genotypes was *stx*_2_ (23/37, 62%), *stx*_1_/*stx*_2_ (13/37, 35%), and *stx*1 (1/37, 3%). Distinct virulence profiles were also found. The most frequent ones were *stx*_1_/*stx*_2_*saa*/*ehxA* (11 strains), *stx*_2_ (11 strains), and *stx*_2_/*saa*/*ehxA* (10 strains).

It is interesting to notice that in one beef retail market three different serotypes were isolated from the same raw ground beef (O8:H19, O79:H19, and ONT:H19), also the virulence profile among ONT:H19 isolates were different between them. From another retail market multiple strains could be isolated belonging to three different serotypes. One strain O41:H14 serotype isolated from the ground beef, one strain O8:H19 serotype isolated from the manipulator's hands and two strains O178:H19 isolated from meat table and knife. Also, the same serotype could be isolated from different sources in the same retail market. For example O157:H7 was isolated from ground beef, mincing machine, table meat, and manipulator's hands at the same retail market, O113:H2 was isolated from ground beef and mincing machine at another retail market, O174:H28 was isolated from knife and meat table at another retail market, and O181:H49 was isolated from ground beef and meat table at another retail market.

## Discussion

The isolation of STEC from foods is problematic because the bacterium is likely to be present in low numbers, may be sublethally injured and is usually accompanied by large population of competent microflora, including other *E. coli*. The IMS improved the isolation sensitivity of O157 strains at least 100-fold (Wright et al., 1994), but the isolation of STEC non-O157 is still a challenge, since non-O157 STEC strains show great genetic and biochemical diversity, and there is no unique phenotypic marker that can differentiate them from other *E. coli*. At the time the study was conducted, there were no standard methods to detect all the serotypes of STEC non-O157 in meat product, so an enrichment protocol originally used for detecting O157:H7 in meat products by USDA-FSIS was adapted to detect STEC in ground beef and environmental samples (USDA-FSIS, 2010). In order to support the growth of all STEC, novobiocin was not added (Vimont et al., 2007; Kanki et al., 2011) and instead, bile salt No. 3 was added to inhibit gram-positive bacteria (Hussein and Bollinger, 2008). Contamination of STEC in ground beef has been studied by researchers in Botswana, Republic of Ireland, Egypt, the Netherlands, Spain, and Canada samples (Heuvelink et al., 1999; Chapman et al., 2001; Cagney et al., 2004; Magwira et al., 2005; Bohaychuk et al., 2006; Mora et al., 2007; Rhoades et al., 2009) and it has been shown that the prevalence of STEC O157:H7 ranges from 0.4 to 3.7%, while STEC non-O157 ranges from 2.4 to 30% (Hussein, 2007). However, studies analyzing meat contact surfaces were not found. In the present study STEC O157 was isolated in 12.2% of ground beef and 2.2% of environmental samples, while STEC non-O157 was detected in 14.4% and 6.6% of the samples, respectively, although Etcheverría et al. (2010) found 40.74% in ground beef. Comparing these results with those reported by other countries as the USA showing that 0.3% of ground beef samples were positive for STEC O157 (Samadpour et al., 2006), or the United Kingdom that reported a 0.8% prevalence analyzing 6303 samples (Chapman et al., 2001), or Ireland with a 2.8% of positive samples from 1533 ground beefs analyzed (Cagney et al., 2004), the positive rates for STEC O157 reported in this study are much higher than the previously described above. A recent study in the USA showed that 7.3% ground beef samples were positive for STEC non-O157 (Bosilevac and Koohmaraie, 2011). In France, 11% of beef samples were contaminated (Mora et al., 2007), while in Australia the positive rate was 16% (Barlow et al., 2006), and was similar to the results obtained in the present study. However, it is very difficult to compare different studies as geographical locations, sampling procedures, isolation, and detection methods are different and can affect the prevalence data significantly.

Screening for the presence of *stx*_1_ and *stx*_2_ by PCR may not have been the best method for the initial determination of prevalence of STEC, since other species of bacteria can possess *stx* genes. The detection of *stx* in samples without performing the corresponding strain isolation is incomplete and is regarded as a presumptive diagnosis, but it is valid for the identification of reservoirs (Scheutz et al., 2001). The difference between O157 and non-O157 isolation rates is notably as observed from isolation results obtained in the present study, STEC O157 could be recovered from approximately the 50% of the beef and 37.5% of environmental samples analyzed, but STEC non-O157 could only be isolated from 27.6% of beef samples and 13.2% of environmental samples. The principal reason is the usage of IMS for O157 strains and the differential phenotypic characteristics that contribute to the identification of these strains. Although Wenting Ju et al. (2012) reported positive rates of 60% using a colony hybridization procedure targeting *stx* genes against 27.6% of isolates obtained in the present work, Bosilevac and Koohmaraie (2011) reported better values using PCR than using colony hybridization. Another difference between non-O157 isolation from ground beef samples and environmental samples lies in the fact that in inert surfaces the bacteria can survive but not multiply, so the number of microorganisms in environmental samples is lower than in the meat, making it harder to be recovered, as it is shown in the present work.

The STEC strains isolated from raw meat did not possess the Locus of Enterocyte Effacement pathogenicity island (LEE PAI) which is most commonly associated with STEC that cause outbreaks and severe disease. Karmali et al. (2003) have classified STEC into seropathotypes according to their relative incidence, frequency of involvement in outbreaks and their association with severe disease. According to such a scheme, excluding O8:H19 and O113:H21, all the STEC non-O157 strains in this study are characterized as isolates that cause low human disease incidence, are rarely associated with outbreaks and do not cause severe human disease. Despite the high prevalence of STEC strains in ground beef, occurrence of human disease is low because most of the strains isolated from food lack adherence factors such as *eae* and *saa*, which contribute to intestinal colonization and therefore the pathogenicity of the strains. STEC O8:H19 and O113:H21 are important serotypes associated with HUS and HC worldwide, including Argentina (Rivas et al., 2011). They were also recovered from ground beef (Bosilevac and Koohmaraie, 2011) and are one of the most frequent serotypes in Argentine cattle (Masana et al., 2011).

Strains belonging to the same serotype could be isolated from different sources of the same retail market; also, the same serotype could be detected in different stores. However, molecular subtyping of the isolates, such as by pulsed field gel electrophoresis (PFGE), should be done to demonstrate possible clonal relatedness.

### Conflict of interest statement

The authors declare that the research was conducted in the absence of any commercial or financial relationships that could be construed as a potential conflict of interest.
